# Congenital Ewing's Sarcoma in a neonate in Uyo - a case report

**DOI:** 10.11604/pamj.2013.15.90.2219

**Published:** 2013-07-09

**Authors:** Frances Sam Okpokowuruk, Iso Oloyede

**Affiliations:** 1 Lecturer/Consultant Paediatrician, University Of Uyo Teaching Hospital, Uyo, Akwa Ibom State, Nigeria

**Keywords:** Congenital Ewing's sarcoma, neonatal period, Nigeria

## Abstract

Congenital Ewing's sarcoma is a very rare occurrence indeed with only one case involving the humerus and none involving the ulna that has been noted in the literature to our knowledge. It is one of those tumours that not only do they rarely occur in the neonatal period, but is also very uncommon in black people. We present a case report of Congenital Ewing's Sarcoma in a black female infant involving the ulna bone and surrounding soft tissues which was diagnosed by histology and immnohistochemistry.

## Introduction

Ewing's sarcoma belongs to the family of tumours referred to as the Ewing sarcoma family of tumours which consists of tumours that have small, round, blue cells [1]. This group of tumours consists of Ewing sarcoma, peripheral primitive neuroectodermal tumor, neuroepithelioma, atypical Ewing sarcoma, and Askin tumor [1]. Ewing's sarcoma is known to be commonest in the teenage years with 64 of the patients presenting in the second decade of life, 27 percent in the first decade and 9 percent in the third decade [2]. Ewing's sarcoma occurring in a neonate is a very rare occurrence in literature.ref. Epidemiologic data also suggest that it is rarely encountered in people of black descent, being found to be nine times more common in whites than in blacks [3, 4]. We report the case of Ewing Sarcoma observed at birth in black infant. It is also less common in females compared to males with a M: F ratio of 1.5:1 [5, 6].

## Patient and observation

Patient N. M. S was a nine week old female infant who presented with a history of a swelling in the left forearm since birth and a 6 week history of fever. The swelling on the left forearm was about the size of a coin at birth and gradually increased in size. At the age of 3 weeks, she was taken to a private hospital where the swelling thought to be a boil was incised and a yellow fluid which was believed to be pus was drained. The swelling thereafter rapidly increased in size with poor wound healing resulting in a large ulcer over the area. There was associated pain as the child was said to cry whenever the swelling was touched. This was said to be after the incision and drainage. There was also limitation of movement at the elbow joint as the swelling spread to involve the elbow joint. A few days after the incision of the swelling, which was 6 weeks before presentation the child developed a low grade intermittent fever which was temporarily relieved by paracetamol. Various herbal preparations were applied to the swelling along with oral administration of over the counter drugs with no improvement. Mother did not book for antenatal care though pregnancy was said to be uneventful with no history of early rash with fever or exposure to irradiation. Mother took herbal preparations for about a month from the 7^th^ month of gestation. She did not take any other known drugs. She delivered via spontaneous vaginal delivery at term at a traditional birth attendant's home.

Physical examination revealed an infant who was mildly pale, afebrile (T = 37°C), anicteric, not cyanosed, and had a left axillary lymphadenopathy measuring 1cm in diameter, firm, mobile and non tender.

Examination of the musculoskeletal system showed a large swelling on the left forearm involving the elbow and upper part of the arm, the overlying skin was hyperaemic and had dilated veins. The swelling which measured 12cm by 10cm was firm, tender with some fluctuant areas. An ulcer measuring 5cm by 8cm was seen on the medial aspect of the extensor surface of the forearm. The edge was raised, the base indurated and the floor had dirty slough.

Other systems were essentially normal. An initial diagnosis of soft tissue tumour probably Rhabdomyosarcoma was made. An initial full blood count done was suggestive of sepsis for which the patient was commenced on intravenous Ceftriaxone, and Gentamicin which were irregular due to fianancial constraints. She also had daily dressing of the ulcer. A week into the admission, a wedge excisional biopsy was done and the histology report showed a small blue cell tumour with the following as possibilities; Desmoplastic small cell tumour, Ewing sarcoma, Lymphoma, and Embryonal Rhabdomyosarcoma. Immunohistochemistry test done at another center using membranous staining with MIC2 (12E7) antigen (CD99) gave a final diagnosis of Ewing's sarcoma. An initial chest x-ray done was normal. X-ray of the left forearm showed erosion of the ulna bone. Other investigations such as wound swab microscopy and culture, blood culture and liver function tests were ordered for but were not done because of financial difficulties. A month after admission, disarticulation of the left arm with an above elbow amputation was performed, and the child was to commence adjuvant chemotherapy with vincristine, doxorubicin, and cyclophosphamide. However, her parents were unable to procure cytotoxic drugs due to financial constraints. On the 48^th^ day of admission, a swelling was noticed over the thoracolumbar spine, which was suspected to be a metastatic lesion. An X-ray of the thoracolumbar spine was requested but was never done due to financial constraints. She continued to have intermittent pyrexia despite treatment with intravenous ceftriaxzone which was later changed to ceftazidime. She also received oral antibiotics when parents could not afford intravenous drugs and antimalarials. She later developed acute watery diarrhea with mild dehydration for which she was given oral rehydration solution. She had a total of three full blood count tests done throughout her hospital stay which were suggestive of sepsis and also electrolytes and urea during the diarrhoeal episode which was essentially normal except for low bicarbonate which was corrected. The patient eventually succumbed to probable sepsis as an immediate cause of death and died after two months on admission.

## Discussion

Ewing's sarcoma is an uncommon tumor especially in the neonatal and infantile age group [5]. In a large series of 734 persons, only 19(2.6 percent) of the total number of cases were found in children below the age of 3years [7]. It is exceedingly rare in the new born [5, 6, 8] with our patient's history dating from birth. It has also been found to be very uncommon in the black race as compared to the Caucasians-our patient was a black female infant. Though it is said to be commoner in males than in females overall, a female predominance has been found in infants and toddlers as was the case with our patient [5, 6, 7]. Although Ewing's sarcoma can arise from any bone in the body, two thirds of the cases have been found in the lower limbs with only 10% involving the upper limbs [8] as was the case with our patient. Involvement of the ulna bone in particular is extremely rare with Paulussen et al [9] finding only 1% of cases to have ulna involvement. To the best of our knowledge, this is the only case of congenital Ewing's sarcoma involving the ulna bone that has been reported in our environment. Cytogenetic and molecular studies are very important as an aid to diagnosis, without which it is impossible to make a conclusive diagnosis of Ewing sarcoma but this modality of investigation was unavailable in our center and indeed, in many hospitals in the developing world. Advanced radiological investigations such as CT scan, MRI could not also be done because of unavailability in our center. Severe financial constraint was a major problem in this patient which not only affected the caliber of investigations that could be done but also therapy as the patient was unable to commence cytotoxics before her demise. An above the elbow amputation was done for the child which was to be followed by chemotherapy with vincristine, doxorubicin and cyclophosphamide. A combination of surgery, radiation and chemotherapy can achieve a 50-70% 5 year survival rate [5, 7, 10] but unfortunately, our patient could not benefit from all the modalities of treatment. The patients’ spent a total of sixty days on admission. Several challenges were encountered in the management of this child such as late presentation by the care givers, lack of laboratory support resulting in a delay in making the diagnosis and financial constraints limiting the scope of investigations and treatment.

## Conclusion

This case presentation high lights a rarely encountered form of bone cancer seen in black people and also in an unlikely age bracket. Therefore clinicians should bear in mind the possibility of this tumor when neonates present with similar findings and take steps to do the necessary investigations. Indeed, the age old adage in medicine that “never say never” still stands.
